# Comparison of the Mental Burden on Nursing Care Providers With and Without Mat-Type Sleep State Sensors at a Nursing Home in Tokyo, Japan: Quasi-Experimental Study

**DOI:** 10.2196/19641

**Published:** 2022-03-23

**Authors:** Sakiko Itoh, Hwee-Pink Tan, Kenichi Kudo, Yasuko Ogata

**Affiliations:** 1 Department of Gerontological Nursing and Healthcare Systems Management Graduate School of Health Care Sciences Tokyo Medical and Dental University Tokyo Japan; 2 Health Services Research and Development Center University of Tsukuba Ibaraki Japan; 3 Department of Genome Informatics Graduate School of Medicine Osaka University Osaka Japan; 4 School of Information Systems Singapore Management University Singapore Singapore; 5 Policy, Research and Surveillance Group, Health Promotion Board Singapore Singapore; 6 Research Innovation Initiatives Organization, Hirosaki University Aomori Japan

**Keywords:** long-term care, caregiver burden, nursing homes, aged, information technology, sensors

## Abstract

**Background:**

Increasing need for nursing care has led to the increased burden on formal caregivers, with those in nursing homes having to deal with exhausting labor. Although research activities on the use of internet of things devices to support nursing care for older adults exist, there is limited evidence on the effectiveness of these interventions among formal caregivers in nursing homes.

**Objective:**

This study aims to investigate whether mat-type sleep state sensors for supporting nursing care can reduce the mental burden of formal caregivers in a nursing home.

**Methods:**

This was a quasi-experimental study at a nursing home in Tokyo, Japan. The study participants were formal caregivers who cared for residents in private rooms on the fourth and fifth floors of the nursing home. In the intervention group, formal caregivers took care of residents who used sleep state sensors on the fourth floor of the nursing home. The sleep state sensors were mat types and designed to detect body motion such as the frequency of toss and turning and to measure heartbeat and respiration. One sensor was placed on a bed in a private room. When body motion is detected, the information is instantly displayed on a monitor at a staff station. In addition, the mental condition of the formal caregivers was measured using a validated self-reported outcome measure—the Profile of Mood States (POMS), Short-Form, 2nd edition. Formal caregivers in both groups received the POMS at baseline, midpoint (week 4), and endpoint (week 8) to identify changes in these domains. The primary outcome was the difference in total mood disturbance (TMD) of the POMS at baseline and week 8.

**Results:**

Of the 22 eligible formal caregivers, 12 (intervention group) utilized sleep state sensors for 8 weeks. The remaining 10 formal caregivers (control group) provided nursing care as usual. As for the primary outcome of the difference between TMD at baseline and week 8, TMD in the intervention group improved by –3.67 versus 4.70 in the control group, resulting in a mean difference of –8.37 (95% CI –32.02 to 15.29; *P*=.48) in favor of the intervention.

**Conclusions:**

The present 8-week study showed that sleep state sensing for elderly residents might not be associated with reduced mental burdens on formal caregivers in nursing homes.

## Introduction

The aging of society is rapidly increasing and expanding in the world. According to the United Nations, there were 703 million persons aged 65 years and older in 2019 worldwide, and the number of older adults is projected to double to 1.5 billion in 2050 [1]. Population aging has been fastest in East and Southeast Asia, Latin America, and the Caribbean [1]. For instance, Japan has the highest proportion of older persons in the world; in 2019, 28.1% of the population (or 35.6 million) was 65 years and older [2]. By 2065, 1 in 2.6 people will be 65 years and older in Japan [2]. Such rapid demographic changes leave countries with insufficient long-term care resources to tackle the challenges associated with an aging population.

In accordance with the aging population in Japan, there has been an exclusive increase in the demand for long-term care [3,4]. The increasing need for long-term care has brought about an increased burden on formal caregivers [5,6]. In particular, the formal caregivers in nursing homes had to deal with exhausting labor (ie, long hours, overtime work, and late-night work). The mental and physical fatigue is severe and also undermining their health, resulting in migraines, depression, and backache [6-9]. To cope with the heavy labor, the Japanese government promoted the utilization of the Internet of Things (IoT) to support formal caregivers [10].

IoT means that everything can be accessed anytime and anywhere, and that applications work without human intervention, as long as there is internet [11]. To date, IoT application studies for health care use include IoT devices for tracking human activities in primary health care centers [12], for medication compliance among older outpatients [13,14], for intensive health guidance among outpatients with diabetes mellitus [15], and for home-based health care [16]. Although research activities on the use of IoT devices to support long-term care for older adults exist, there is limited evidence on the effectiveness of these interventions among formal caregivers in nursing homes [17-19]. In this study, we investigate whether sleep state sensors for supporting long-term care can reduce the mental burden of formal caregivers in a nursing home.

## Methods

### Study Design and Participants

We conducted a quasi-experimental study to examine the effects of sleep state sensors for supporting formal caregivers at a nursing home. The participants were eligible if they were formal caregivers, aged at least 20 years, and worked at a nursing home. Participants are excluded from the study if they plan to leave the job within 8 weeks. In this study, we investigated whether long-term care for residents using mat-type sleep state sensors that detect the resident’s sleep state reduces the caregiver’s mental burden compared with usual care.

### Procedures

**Figure 1:**
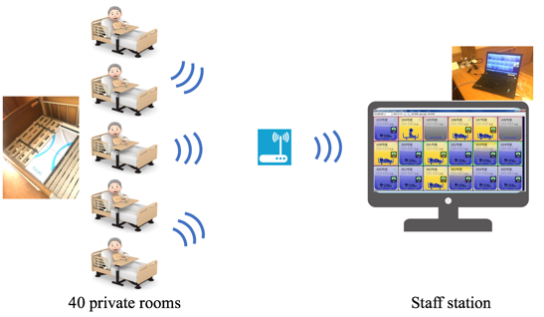
Overview of the sleep state sensors.

An intervention group received sleep state sensors to provide long-term care for residents in all 40 private rooms on the fourth floor of a nursing home in Zenkoukai, Tokyo. The sleep state sensors were mat types and designed to detect body motion such as the frequency of toss and turning and to measure heartbeat and respiration. One sensor was placed on a bed in a private room. When body motion is detected, the information is instantly displayed on a monitor at a staff station. The monitor showed the sleep state (ie, awake or asleep) and action state (ie, lying, sitting, or leaving bed) of the residents at all times. When formal caregivers cared for residents in each private room, they checked the sleep and active state of the resident on the monitor and visited the private room (Figure 1). For instance, in the intervention group, when the visiting the room for elimination care, the formal caregiver checked the resident’s sleeping or waking status on display and visited the room for elimination care when the resident was awake as much as possible. By contrast, formal caregivers in the control group (on the fifth floor of the nursing home) provided long-term care for residents as usual.

The sensors were expected to allow the formal caregivers to check the status of each resident in each private room of nursing homes in real time on PCs at the staff station, thus enabling them to provide care efficiently. Furthermore, by accumulating these records, it was possible to understand the rhythm of each resident’s life. As a result, it was expected that care plans can be formulated to match the rhythm of each resident’s life.

In addition, the mental condition of the formal caregivers was measured using self-reported outcome measures, the Profile of Mood States (POMS), Short-Form, 2nd edition (POMS 2) [20,21]. POMS 2 was published in 2012 to assess transient feelings and mood, and has already been validated by Heuchert and McNair [20]. The Japanese version of the POMS 2 scale has already been validated for reliability and validity [22,23]. Participants in both groups received the POMS at baseline, midpoint (week 4), and endpoint (week 8) to identify changes in these domains. The POMS assessed mood states of individuals, or transient, fluctuating feelings and enduring affect states [20,21].

### Outcome

The primary outcome was the difference at 8 weeks in the total mood disturbance (TMD) of the POMS. Referring to previous studies, we set the duration of the sensor-based intervention at 8 weeks [24,25]. The TMD indicated the extent to which formal caregivers experienced overall negative or positive affect, or the degree of overall mood disorder, where a higher score is indicative of the bad mood. The TMD was a composite score of 5 negative mood states (ie, anger-hostility, confusion-bewilderment, depression-dejection, fatigue-inertia, tension-anxiety) and a positive mood state (ie, vigor-activity). As for the TMD and negative mood states, higher scores could indicate a problem. Regarding the positive mood states, lower scores indicate a problem. Regarding clinically significant differences, we considered a difference of 8 points or more in the TMD as clinically significant, referring to previous studies [26]. The secondary outcome was the change at 3 time points (ie, baseline, week 4, week 8) of the TMD to clarify the change of the mental burden immediately after the introduction.

### Statistical Analysis

For the primary analysis, we performed an analysis of covariance (ANCOVA), which is a blend of analysis of variance (ANOVA) and general linear regression to evaluate differences between groups. We conducted the ANCOVA using POMS 2 (eg, TMD), intervention, measurement timing, and an interaction term between intervention and measurement timing. For secondary outcomes, the difference between the intervention and control groups was analyzed using ANCOVA. For the PMD at the baseline, midpoint (4 week), and endpoint (8 week), we used a repeated ANOVA to assess the change of TMD in the intervention and control groups. A *P* value less than .05 was considered statistically significant. Statistical analysis was performed using Stata, version 16.0 (StataCorp).

### Ethical Approval

Medical ethical approval was obtained from the Medical Ethical Committee of Tokyo Medical and Dental University (M2017-228-2). All participants gave written consent for participation in the study.

## Results

A total of 25 formal caregivers were recruited; however, 1 formal caregiver in the intervention group and 2 formal caregivers in the control group discontinued because of leaving the job at the nursing home. Among the 22 formal caregivers, the median age was 31 years (IQR 28-37 years); 9 (41%) formal caregivers were women versus 13 (59%) men. The median working period at the nursing home was 59 months (IQR 14-92 months). Among the 22 formal caregivers, 12 (55%) were certificated care workers (ie, those who have national qualifications). Tables 1 and 2 present the baseline characteristics of the formal caregivers and older persons, respectively, by the intervention and control groups.

**Table 1:** Baseline characteristics of the formal caregivers.

Population			Intervention group (n=12)		Control group (n=10)		*P* value
Age (years), median (IQR)			31 (28-32)		32 (30-55)		.27^a^
**Sex, n (%)**							.19^b^
	Women	3 (25)		6 (60)			
	Men	9 (75)		4 (40)			
Working period^c^ (months), median (IQR)			57 (7-74)		76 (34-93)		.31^a^
**Certifications, n (%)**							.85^b^
	Certificated care worker	7 (58)		5 (50)			
	Other	2 (17)		1 (10)			
	None	3 (25)		4 (40)			

^a^Mann-Whitney *U* test.

^b^Fisher exact test.

^c^Working period at the nursing home.

**Table 2:** Baseline characteristics of the older persons.

Population			Intervention group (n=40)		Control group (n=40)		*P* value
Age (years), median (IQR)			87 (82-91)		87 (82-91)		>.99^a^
**Sex, n (%)**							.79^b^
	Women	32 (80)		30 (75)			
	Men	8 (20)		10 (25)			
**Care need levels, n (%)**							.81^b^
	1	0 (0)		0 (0)			
	2	0 (0)		1 (3)			
	3	9 (23)		9 (23)			
	4	15 (38)		17 (43)			
	5	16 (40)		13 (33)			

^a^Mann-Whitney *U* test.

^b^Fisher exact test.

Among the 22 formal caregivers, 12 were assigned to the intervention group, and 10 to the control group. Out of the 12 formal caregivers in the intervention group, 50% (n=6) experienced positive TMD at the endpoint (ie, week 8). Of the 10 formal caregivers in the control group, 50% (n=5) also experienced positive TMD at the endpoint (ie, week 8). As for the primary outcome of the difference between TMD at baseline and week 8, TMD in the intervention group improved by –3.67 versus 4.70 in the control group, resulting in a mean difference of –8.37 (95% CI –32.02 to 15.29; *P*=.48) in favor of the intervention. Although significant differences were not observed (see above) between the intervention and the control groups, the sign of regression coefficients was negative.

Table 3 shows the effect of the intervention on secondary outcomes. The change in anger-hostility from baseline to 8 weeks in the intervention group improved by –0.84 versus 0.90 in the control group, resulting in a mean difference of –1.73 (95% CI –7.43 to 3.96; *P*=.54; Tables 1 and 2). The change in depression-dejection in the intervention group improved by –0.16 versus 1.60 in the control group, resulting in a mean difference of –1.77 (95% CI –6.49 to 2.96; *P*=.45). The change in tension-anxiety in the intervention group improved by –0.91 versus 0.20 in the control group, resulting in a mean difference of –1.12 (95% CI –6.58 to 4.34; *P*=.68). Significant differences were not observed (see above) in 5 negative mood states (ie, anger-hostility, confusion-bewilderment, depression-dejection, fatigue-inertia, tension-anxiety) and a positive mood state (ie, vigor-activity).

**Table 3:** Mean difference in change of the Profile of Mood States.

Parameter	Baseline^a^		Week 8^a^		Mean difference in change (intervention vs control) (95% CI)	*P* value^b^
	Intervention (n=12)	Control (n=10)	Intervention (n=12)	Control (n=10)		
Total mood disturbance	27.67 (25.27)	25.9 (13.55)	24.00 (19.29)	30.60 (9.92)	–8.37 (–32.02 to 15.29)	.48
Anger-hostility	5.67 (5.43)	6.00 (4.69)	4.83 (4.41)	6.90 (2.95)	–1.73 (–7.43 to 3.96)	.54
Confusion-bewilderment	7.58 (5.82)	5.00 (3.26)	7.42 (3.84)	6.00 (2.86)	–1.17 (–6.42 to 4.09)	.66
Depression-dejection	6.33 (4.29)	4.60 (2.73)	6.17 (4.08)	6.20 (3.46)	–1.77 (–6.49 to 2.96)	.45
Fatigue-inertia	8.17 (5.92)	9.60 (4.13)	6.92 (5.77)	9.70 (3.85)	–1.35 (–7.78 to 5.08)	.67
Tension-anxiety	8.83 (5.52)	7.80 (3.57)	7.92 (4.17)	8.00 (3.52)	–1.12 (–6.58 to 4.34)	.68
Vigor-activity	8.92 (4.89)	7.10 (4.06)	9.25 (4.90)	6.20 (4.77)	1.23 (–4.71 to 7.18)	.68

^a^Mean (standard error).

^b^Analysis of covariance.

**Figure 2:**
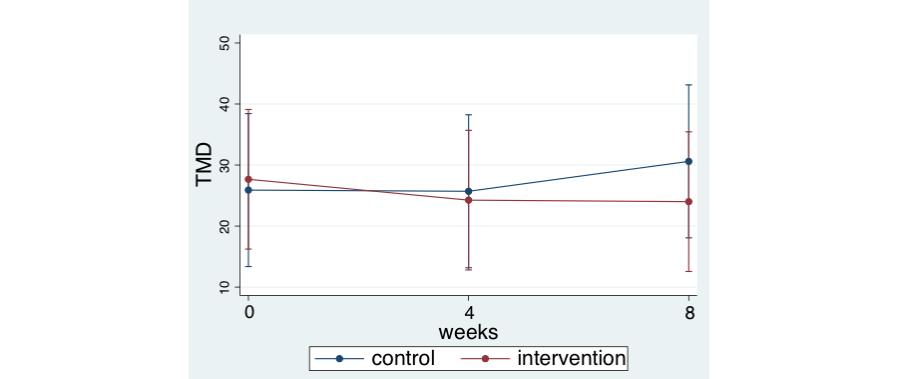
Change of the TMD from baseline to week 8. TMD: total mood disturbance.

For the PMD at the baseline, midpoint (4 weeks), and endpoint (8 weeks), we used a repeated ANOVA to assess the change of TMD in the intervention and control groups. The results indicated that the mean differences (intervention vs control) at the baseline, midpoint, and endpoint were 1.77 (95% CI –15.20 to 18.73; *P*=.84), –1.45 (95% CI –18.42 to 15.52; *P*=.87), and –6.6 (95% CI –23.57 to 10.37; *P*=.44), respectively (Figure 2).

## Discussion

### Principal Findings

We investigated whether sleep state sensors for supporting long-term care can reduce the mental burden of formal caregivers in a nursing home. The primary outcome was the difference from baseline to endpoint (week 8) on the TMD of the POMS, or the degree of overall mood disorder. As the result of this study, the TMD in the intervention group improved by –3.67 versus 4.70 in the control group, resulting in a mean difference of –8.37 (95% CI –32.02 to 15.29; *P*=.48). No significant difference was observed in the study.

In contrast to previous studies with sensors [27-29], utilization of mat-type sleep state sensors was not associated with improving burdens of formal caregivers in the nursing home. Notably, the scales to measure mental condition in the previous study were different from those used in this study; for example, the Satisfaction with Work Questionnaires, the Satisfaction with Life Scale [30], and the Life Satisfaction Questionnaire were used previously [31]. One possible reason for the lack of association between utilization of sleep state sensors and TMD of formal caregivers may be time-lag bias; that is, the impact of using sleep state sensors might appear much later. In our study, the mean differences in change (intervention vs control) at baseline, midpoint, and endpoint were 1.77 (95% CI –15.20 to 18.73; *P*=.84), –1.45 (95% CI –18.42 to 15.52; *P*=.87), and –6.6 (95% CI –23.57 to 10.37; *P*=.44), respectively. Although significant differences were not observed, the mean differences in change (intervention vs control) increased gradually. Thus, further studies over a longer duration would be needed to investigate the association [29, 32, 33].

As for the 5 negative mood states (ie, anger-hostility, confusion-bewilderment, depression-dejection, fatigue-inertia, and tension-anxiety) and a positive mood state (ie, vigor-activity), the greatest change in the intervention group occurred in the fatigue-inertia category. By contrast, there was little change in the fatigue-inertia category in the control group. The use of sensors may therefore be related to reducing fatigue. In future studies, we need to research the differences in the changes in each item.

According to an interview with some formal caregivers in the intervention group, the timing of checking the residents’ condition on the monitor was when they did routine rounds to check on the residents’ health at night, and when they provided excretory care during the day. They then adjusted the time of care when the resident was asleep. Thus, change in the timing of care provision might enhance the quality of care and improve the satisfaction of residents [34-36]. In addition, it was expected to be useful for countermeasures against infectious diseases because the residents’ condition can be ascertained without visiting the room. Further studies about enhancing the quality of care and combating infectious diseases are needed to investigate the effect of the intervention. Moreover, further research on the mechanism of how sensor use would impact care is needed.

### Limitations

Several limitations of this study need to be noted. First, the participants were selected from just 1 nursing home in Tokyo, Japan. Thus, our results cannot be generalized because selection bias may be present. Second, random assignment could not be performed, which may have caused selection bias. Third, the small sample size of this study must be noted. Fourth, the follow-up period might be insufficient, and we did not take into account the time-lag bias. Finally, we cannot completely eliminate the effects of potential confounding factors, including socioeconomic status and educational status of formal caregivers. However, to the best of our knowledge, this is the first study to explore the impact of mat-type sleep state sensors on formal caregivers in a nursing home in Japan, which would contribute to the development of future research on long-term care.

### Conclusions

For an 8-week study in a nursing home, sleep state sensing for elderly residents might not be associated with reduced mental burdens on formal caregivers. The findings imply that further studies over a longer duration would be needed to investigate the association between the utilization of mat-type sleep state sensors and reduced mental burdens on formal caregivers in nursing homes.

## Abbreviations

ANCOVA: analysis of covariance
ANOVA: analysis of variance
IoT: internet of things
POMS: Profile of Mood States
TMD: total mood disturbance
